# ‘We have so much to offer’: Community members’ perspectives on autism research

**DOI:** 10.1177/13623613241248713

**Published:** 2024-05-13

**Authors:** Tori Haar, Charlotte Brownlow, Gabrielle Hall, Melanie Heyworth, Wenn Lawson, Rebecca Poulsen, Tamara Reinisch, Elizabeth Pellicano

**Affiliations:** 1 Macquarie University, Australia; 2 Cooperative Research Centre for Living with Autism (Autism CRC), Australia; 3 Reframing Autism, Australia; 4 University of Southern Queensland, Australia; 5 ORIMA Research, Australia; 6 University College London, UK

**Keywords:** engagement, neurodiversity, participatory research, translational research

## Abstract

Autism research is on the cusp of significant change. There are mounting calls from Autistic self-advocates, researchers, and other scientists for a paradigm shift in autism research. Yet little is known about what non-academic members of the Autistic and autism communities think about autism research. We – a team of Autistic and non-autistic researchers – addressed this gap by conducting a secondary analysis of data collected as part of an in-depth research priority-setting exercise in Australia. Specifically, we asked: what are community members’ views and experiences of autism research? In immersive online text-based focus-group discussions held over 10 days, 55 community members, including Autistic people, family members, and professionals, shared their perspectives and experiences of autism research. We analysed the data using reflexive thematic analysis, adopting an inductive approach. Respondents resoundingly agreed that autism research could play a powerful role in helping to shape good Autistic lives, yet they felt that to do that effectively, major changes were needed. These included a shift away from overly narrow, deficits-based views of Autistic people to a greater focus on contextual factors, and more community-led decisions in what is researched and how. These findings highlight new possibilities for research processes, findings, and translation.

**Lay Abstract:**

Autism research is changing. Autistic activists and researchers want Autistic people in the community to have more of a say about what is researched and how. But we haven’t asked people in the community what they think. This study used the information obtained from 55 community members, including Autistic people, their families, and professionals working with Autistic people, from an existing study on their priorities for autism research. We re-looked at what was said to see if we could understand community members’ views and experiences of autism research. People agreed strongly that research can play a powerful role in shaping good Autistic lives. They also felt that big changes were needed for research to do this. Some of these changes were that researchers should stop thinking about autism narrowly and in a negative way, where Autistic people are seen as the problem. Researchers need to think more about how to improve systems, experiences and how other people respond to Autistic people. They also want the autism community to be more involved in what is researched and how it is researched. The findings from our study here highlight the potential for research to be positive when Autistic people and their families are listened to, approached with understanding, and are respected and valued as individuals in the research process.

Autistic people deserve to live healthy and fulfilling lives of their own design. Yet, despite enormous advances in autism science, the lives of Autistic^1^ people remain profoundly difficult. Autistic children, young people and adults experience intergenerational inequalities – in life expectancy, physical and mental health, education, employment, and social life (see Pellicano, Hall, et al., 2022, for review). Many in the autism science community are committed to tackling these inequalities and some of the very best genetic and neurobiological research in the world is directed towards autism (G. Dawson, 2013). Nonetheless, Autistic experiences and the inequalities and injustices faced by Autistic people have largely not changed, despite substantial investment from both governments and philanthropic foundations (Office of Autism Research Coordination (OARC), National Institute of Mental Health, on behalf of the Interagency Autism Coordinating Committee, 2019) and extensive scientific productivity (OARC, 2012; Rong et al., 2022).

As a result, there are increasing suggestions that greater efforts should be made to connect research to the complex reality of Autistic people’s everyday lives (Pellicano, Hall, et al., 2022). This in part echoes a broader trend towards co-design and co-production in research, whereby academic researchers cede some power and responsibility to lay communities in order more closely to reflect everyday concerns and experiences (Nature, 2018). Some researchers, both Autistic and non-autistic researchers, have gone further still and adopted the neurodiversity paradigm (Kapp, 2020; Pellicano & den Houting, 2022; Walker & Raymaker, 2020). This has led to a critique of dehumanising, ableist language and discourse on autism (e.g. Botha, 2021; Bottema-Beutel et al., 2021; Gernsbacher, 2017; Kapp, 2023; Milton & Bracher, 2013; Natri et al., 2023); a new emphasis on services and supports that enable Autistic flourishing (Chapman & Carel, 2022; Ne’eman, 2021; Pellicano & Heyworth, 2023); a new set of research priorities stressing issues of immediate and everyday concern (OARC, National Institute of Mental Health, on behalf of the Interagency Autism Coordinating Committee, 2019; Pellicano et al., 2013; Roche et al., 2021); and a concerted effort to draw on the insights of Autistic people (Jaswal & Akhtar, 2018; McGeer, 2004; Milton, 2012; Milton & Bracher, 2013) and for Autistic scholars themselves to lead such research (Dwyer, 2022; Poulsen et al., 2023).

Encouragingly, there have been some significant changes as a result of these pressures (Fletcher-Watson et al., 2019; Nicolaidis et al., 2011; Pellicano, 2020; Pellicano & Stears, 2011). These efforts have included bringing together non-academic community members to shape the research agenda (Pellicano et al., 2014b; see Roche et al., 2021, for review). In Australia – the context of the current study – this community involvement has shifted the funding landscape, from a preponderance of funding allocated to biomedical research to a more even distribution of funding – on biological research *and* topics prioritised by the Autistic community, which tend to be those which focus on issues that make a real and immediate difference to everyday life (den Houting & Pellicano, 2019).

While community priority-setting exercises are important, on their own they tell us little about community members’ current experiences of autism research, and especially their views on *how* autism research, of whatever kind, should be conducted. One key study conducted a decade ago in the United Kingdom reported overwhelmingly negative experiences of research (Pellicano et al., 2014a). Family members felt frustrated at being ‘mined’ for information about themselves and/or their child, with little-to-no feedback provided by researchers, and therefore little opportunity to learn about the research results and what they might mean for them. Similarly, Autistic adults reported feeling ‘objectified’ by ‘dehumanising’ interactions, with their experiential expertise perceived to be disregarded by researchers. Some felt ‘let down’. More recent perspective pieces by Autistic people have reinforced these views (Botha, 2021; Lory, 2019; Luterman, 2019; Michael, 2021).

## The current study

The current study sought to understand Autistic and autism community members’ views and experiences of autism research elicited during a priority-setting exercise commissioned by the Australasian Autism Research Council (AARC). The AARC, which sits under the auspices of the Cooperative Research Centre for Living with Autism (Autism CRC), includes representatives of the Autistic and broader autism communities, including Autistic people, family members, service providers, health and education professionals, government employees, and researchers.

In 2019, the AARC conducted its first community consultation (*n* = 1102), identifying 10 broad research priority areas for Australian autism research (AARC, 2019). In 2020, the AARC commissioned ORIMA Research to conduct in-depth online focus groups, to identify more detailed research questions within five of the ten research priority areas, including Education; Health and Wellbeing; Employment; Justice^2^; and Communication (AARC, 2021). Focus group members drew on their own professional, personal and/or lived experience to inform a list of research topics and questions within each Priority Area.

During these discussions, focus-group members were also invited to discuss their perspectives on, and experiences of, autism research. This article is drawn from our secondary analysis of that focus group data as we sought to discover Australian community members’ views and experiences of autism research.

## Method

### Recruitment

The recruitment and selection of focus-group members was conducted by the AARC through an Expression of Interest (EOI), to ensure diversity of experience and expertise (Pratt, 2021). It was advertised online through the Autism CRC and the AARC members’ community connections. Each group was required to include at least three Autistic people, two parents/carers/family members, and two relevant professionals. EOIs were particularly encouraged from Autistic people who predominantly communicated in a non-traditional way (e.g. using Augmentative and Alternative Communication), people living in regional or remote areas, and/or those from Australia’s indigenous population.

### Participants

EOIs were received from 70 interested community members, and of those, 55 (78%) participated in the consultation (see Table 1). They came from all over Australia and ranged in age from 18 to 59 years. They often reported multiple links to the Autistic and autism communities (e.g., an Autistic person, also a caregiver to an Autistic person and a professional working in a relevant field): 33 people (60%) identified as Autistic, 41 (74%) as a parent/carer of an Autistic child and/or adult, and 18 (33%) as a relevant professional. Only seven people (13%) reported no personal connection to autism.

**Table 1.** Respondents’ demographic information, by priority area.

	Communication (*n* = 12)	Education^a^ (*n* = 11)	Employment (*n* = 12)	Health & Wellbeing (*n* = 10)	Justice (*n* = 10)	Total (*n* = 55)
**Age range**						
18–29 years	3 (25%)	2 (18%)	1 (8%)	0	0	**6 (11%)**
30–39 years	3 (25%)	4 (36%)	3 (25%)	3 (30%)	2 (20%)	**15 (27%)**
40–49 years	3 (25%)	4 (36%)	5 (42%)	4 (40%)	6 (60%)	**22 (40%)**
50–59 years	2 (17%)	1 (9%)	3 (25%)	3 (30%)	1 (10%)	**10 (18%)**
Prefer not to say	1 (8%)	0	0	0	1 (10%)	**2 (4%)**
**Pronouns**						
She/her	8 (67%)	9 (82%)	9 (75%)	8 (80%)	9 (90%)	**43 (78%)**
He/him	3 (25%)	0	1 (8%)	1 (10%)	1 (10%)	**6 (11%)**
They/them	1 (8%)	0	2 (17%)	0	0	**3 (5%)**
Other/multiple pronouns	0	2 (18%)	0	0	0	**2 (4%)**
Prefer not to say	0	0	0	1 (10%)	0	**1 (2%)**
**Australian State/Territory**						
New South Wales	5 (42%)	4 (36%)	3 (25%)	5 (50%)	4 (40%)	**21 (38%)**
Victoria	4 (33%)	2 (18%)	5 (42%)	2 (20%)	1 (10%)	**14 (25%)**
Queensland	1 (8%)	3 (27%)	3 (25%)	2 (20%)	2 (20%)	**11 (20%)**
South Australia	1 (8%)	0	0	0	3 (30%)	**4 (7%)**
Western Australia	1 (8%)	1 (9%)	1 (8%)	1 (10%)	0	**4 (7%)**
Australian Capital Territory	0	1 (9%)	0	0	0	**1 (2%)**
**Regionality** ^b^						
Urban	12 (100%)	9 (82%)	11 (92%)	7 (70%)	9 (90%)	**48 (87%)**
Regional	0	2 (18%)	1 (8%)	3 (30%)	1 (10%)	**7 (13%)**
**Language other than English spoken at home**	2 (17%)	1 (9%)	1 (8%)	1 (10%)	1 (10%)	**6 (11%)**
**Uses non-traditional forms of communication**	2 (17%)	0	1 (8%)	0	0	**3 (5%)**
**Personal connection to autism** ^c^						
Autistic	4 (33%)	4 (36%)	10 (83%)	9 (90%)	6 (60%)	**33 (60%)**
Parent of an Autistic child	6 (50%)	7 (64%)	6 (50%)	9 (90%)	4 (40%)	**32 (58%)**
Parent of an Autistic adult	2 (17%)	1 (9%)	1 (8%)	1 (10%)	4 (40%)	**9 (16%)**
Other relative	0	0	3 (25%)	2 (20%)	0	**5 (9%)**
None	2 (17%)	3 (27%)	0	0	2 (20%)	**7 (13%)**
**Relevant professional** ^d^	2 (17%)	4 (36%)	2 (17%)	4 (40%)	6 (60%)	**18 (33%)**

a One member of the Education Priority Area identified as being from Aboriginal and Torres Strait Islander background.

b Determined using the Australian Statistical Geography Standard Postcode Concordance (2016) – postcodes were only collected for community members who identified themselves as coming from a regional or remote area.

c Respondents were able to select all options that applied to them; therefore, percentages do not add up to 100.

d Eighteen professionals (33%) took part, including a speech pathologist and early-childhood educator (Communication); teachers and special educators (Education); support coordinator and employer (Employment); nurses, psychologist and social worker (Health and Wellbeing); psychologists, social worker, police officer, lawyer and support worker within the criminal justice system (Justice).

### Procedure

Respondents took part via a 10-day online discussion board, one for each Priority Area. These were conducted in September–October 2020 and moderated by an Autistic advocate and researcher (G.H.) and a trained nonautistic facilitator (overseen by T.R.) at ORIMA Research. The discussion boards facilitated discussion of respondents’ views and perspectives of current and future autism research. They began with broad questions about their own connection to the Autistic and autism communities, to provide context. Respondents were then invited to discuss the challenges and strengths for the Autistic and autism communities in their respective Priority Area. The discussions often extended to autism support practices, not solely research. Finally, respondents were asked to identify research questions they wanted to see prioritised (see AARC, 2021, for full details).

Ethical approval for this study was granted by ORIMA Research Human Research Ethics Committee approval number: 0062020. All data were de-identified prior to the current analysis.

### Data analysis

Our interdisciplinary team brought together perspectives from psychology (E.P., C.B., T.H. and W.L.), neuroscience (R.P.), education (E.P., M.H.), disability studies (T.H.), mental health nursing (G.H.) and social policy (T.R.). Interpretation of the data was further enriched by team members’ relevant positionalities as Autistic researchers, parents of Autistic children/adults and as prominent advocates in the Autistic community (T.H., G.H., M.H., W.L. and R.P.), their alliance with the neurodiversity paradigm (Chapman & Botha, 2022; Pellicano & den Houting, 2022) and the social model of disability (Oliver, 1996), and their commitment to, and advocacy for, community involvement in autism research. We acknowledge that – in the spirit of reflexive thematic analysis (Braun & Clarke, 2019) – these particular values and assumptions will be reflected to some extent in our findings.

We followed Braun and Clarke’s (2019) method for reflexive thematic analysis within an essentialist framework. We identified themes using an inductive approach to identify patterned meanings within the dataset. Two authors, one Autistic, one non-autistic (T.H. and E.P.), immersed themselves in the data, closely reading and re-reading the transcripts from each Priority Area discussion board and taking reflexive notes on striking and recurring observations. After discussion of potential codes, our Autistic co-author, TH, led the analysis, applying codes to each transcript (managed in NVivo, version 12). She discussed the codes and resulting observations with E.P. at multiple points during this stage of the analysis, and re-worked codes, as appropriate. Next, T.H. and E.P. worked together to group codes to identify candidate themes and subthemes. T.H. then generated a draft thematic map, and the relevant data were collated under each theme and subtheme, with discussion and guidance from E.P. The draft analysis was then discussed, reviewed and finalised with the broader team, focusing on semantic features of the data. Analysis was therefore iterative and reflexive, with themes and subthemes identified through systematic engagement with the data combined with an active and deeply reflexive approach to analysis, influenced by the researchers’ own aims, positionalities and interpretation of the data.

### Community involvement

The current study involved five Autistic (T.H., G.H., M.H., W.L. and R.P.) and three non-autistic (E.P., C.B. and T.R.) researchers, all of whom had worked together previously in some way, including as part of the AARC and some in long-standing collaborations. One Autistic researcher (G.H.) had also been involved as an ‘insider researcher’ in the primary research, moderating the original online discussion boards – a methodological feature demonstrated to enhance the effectiveness of qualitative autism research (Pellicano, Lawson, et al., 2022).

The research process entailed multiple meetings and email conversations to identify, and agree on, the research question and, subsequently, to formally request access to the original data from the AARC (one Autistic and non-autistic researcher led the data request). Once the data had been secured, Autistic author (T.H.) led the analysis, reading through the discussion group transcripts and meeting frequently with non-autistic researcher (E.P.) to discuss their content as part of the analytic process. All team members met regularly to reflect thoroughly upon the analysis, which resulted in changes in the thematic structure and individual theme/subtheme labels. T.H. and E.P. led on the writing of the manuscript, and everyone contributed to the final manuscript.

We adopted a proactive and collaborative approach to address potential power imbalances with our team. This included communicating openly about the research goals and procedures, as well as the expectations and responsibilities for team members regarding the analysis and writing process. We also sought to cultivate a safe and supportive space during meetings and email conversations to ensure that all members of the team felt that they could express their views and that these views would be heard. These efforts resulted in a genuinely collaborative partnership and further reinforced team members’ strong, trusting relationships that predated this particular study.

## Results

**Figure 1.**
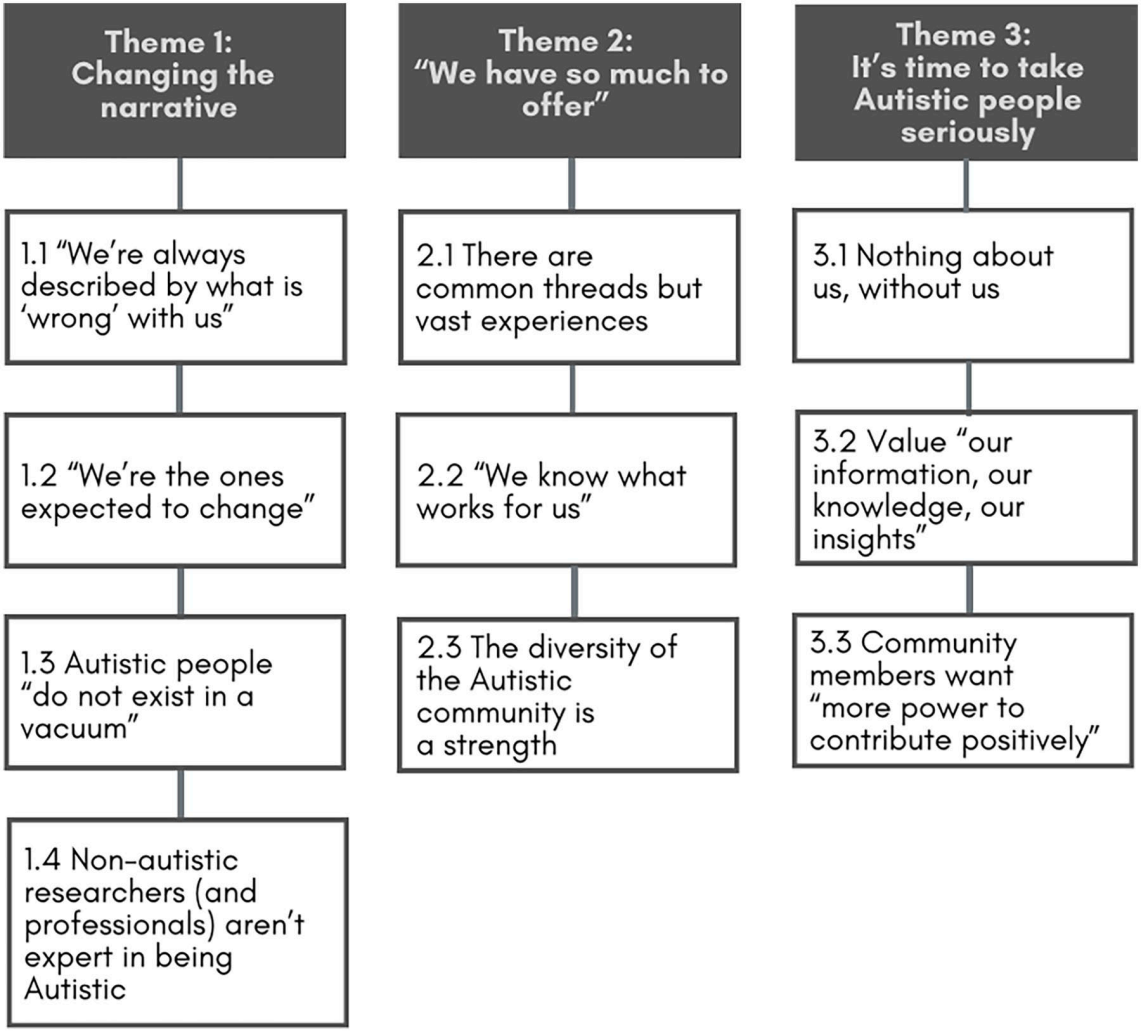
Themes and subthemes identified from respondents’ discussions.

We identified three key themes in our community respondents’ comments when discussing their priorities for autism research (Figure 1). These themes were broad, drawing both on concerns about the perception of autism in society reflected in the research process *and* on specific concerns relevant only to the conduct of research. The close interaction between these different kinds of concerns made it crucial to include both. Quotes are attributed via Priority Area (COM: Communication; EDU: Education; EMP: Employment; H&W: Health and Wellbeing; JUST: Justice).

### Theme 1: changing the narrative

#### Subtheme 1.1: ‘we’re always described by what is “wrong” with us’

Respondents were clear that the prevailing narrative on autism was overwhelmingly negative. According to some, it needed to be ‘dismantled’ entirely (COM). They felt there was an inherent ‘assumption that the default way of being in this world is a neurotypical one’ (COM), which meant that ‘we’re always described by what is “wrong” with us’ (COM), as ‘disordered neurotypical people rather than normal, healthy Autistic people’ (EMP). This negative discourse was perceived to have far-reaching consequences – for the person themselves and for those supporting them.

Respondents observed how this deficit-focused framing starts early in an Autistic child’s life, where ‘even children’s play is pathologized . . . we do “it” wrong’ (H&W), which they felt negatively influences parents’ perceptions of their children. They also described how these negative perceptions continue into school, where the often-pervasive use of the language of ‘disorder’ ‘implies we are broken’ (EDU) and that being Autistic was perceived to be used as ‘a blanket explanation for behaviour and personality . . . which compounds stigma, stereotypes, and preconceptions of “less than” or “abnormal”’ (EDU). Respondents also reported how such negative framings extended into the world of work: ‘if all you are exposed to is a narrative of your “deficits,” it will be hard to see your strengths’ (EMP), which are often ‘only being recognised when they [Autistic people] are perceived to be “inspiring”’ (EMP).

While respondents recognised that Autistic people have a complex array of characteristics, including both strengths and challenges, they felt the challenges could be overemphasised, mis-attributed (‘we can find aspects of daily living challenging, but that doesn’t have to mean that WE are challenging’; JUST), or all-too-easily dismissed within the current deficit-based framework: ‘you can’t tell how disabled we are by looking at us or by spending 10 minutes with us’ (H&W). Ultimately, being told they are ‘disordered’ was felt to have a detrimental impact on Autistic people’s sense of self ( ‘it took me a long time to start to build self-esteem and pride in my autistic identity – a big part of that is because of the framing of autism as lesser’; COM) and their mental health ( ‘I carry around a lot of trauma from these things’; COM).

#### Subtheme 1.2: ‘we’re the ones expected to change’

According to respondents, one key consequence of this pathologising narrative was that any deviation from neurotypical is often taken to be ‘wrong and in need of fixing and changing’ (COM). The onus, therefore, was all-too-often on the individual to modify or inhibit how they interact with the people around them. Respondents often spoke of how this played out at school: ‘When [child] wouldn’t comply with teacher directions, such as “please do X” (which we know is a sensory trigger), their response was “he needs more therapy” or ‘you need to review his medication’’ (EDU). Indeed, many felt that inclusion of Autistic children at school was often ‘allowed on the condition (generally unspoken, even concealed) that behavioural modification by the child is compulsory’ (H&W). They also emphasised how children were often acutely aware of others’ perceptions that ‘they are broken’ (EDU), as one teacher explained, ‘I once had an 8 year-old say to me, “Mummy loves me, but she makes herself very tired driving me to different teachers all the time. I hope they can fix me, so Mummy doesn’t have to try so hard to love me”’ (EDU).

Some respondents were frustrated by the pervasive expectation of having to change ‘intrinsically autistic behaviours to fit in (e.g. eye contact, stimming, speaking)’ (COM). They stressed that the ‘energy spent conforming to what we believe is “expected” of us socially’ meant that ‘we are not presenting our authentic selves and we exhaust ourselves and this is what leads to our high levels of depression and suicide’ (JUST). Some were pessimistic that their efforts could ever be enough: ‘I’ve spent years trying to change for everyone else, trying to find ways to be, to communicate, to look, to *exist*, that non-autistic people might accept, and it’s never worked’ (COM). Instead, respondents wanted greater awareness of the efforts Autistic people make for social interactions or to complete everyday tasks: ‘I don’t think a lot of people outside of the autistic community realise the planning and preparation that goes into our lives’ (H&W).

#### Subtheme 1.3: Autistic people ‘do not exist in a vacuum’

Respondents were also frustrated about the overemphasis on the Autistic individual themselves, rather than the broader relational and societal contexts of people’s lives. They were emphatic that Autistic people ‘do not exist in a vacuum’ (H&W) and shared many examples of inaccessible and inhospitable interactions, environments, and systems. They wrote of how ‘many of our health problems begin with poor environmental fit’ which can mean ‘the so-called support services compound the problems’ (H&W). This issue also appeared to be particularly acutely felt in the education system, being a system that people encounter early in their lives as they are critically building identity and framing how they fit into the world. One person described: ‘Our current system just doesn’t cope with “difference”, and autism presents a whoooooole bunch of difference that the system (and all of its component parts) just can’t seem to compute or accommodate’ (EDU). Respondents highlighted a lack of consistency in approaches to learning and behavioural management and teachers’ limited knowledge of how best to support their Autistic students: ‘I was shocked the school didn’t really know what to do’ (EDU). Respondents therefore wanted ‘a better understanding of autistic challenges that could help non-autistic people meet us half-way, and help create more inclusive environments’ (EDU).

The importance of context and of meeting Autistic people half-way was emphasised by respondents across Priority Areas. They wanted greater acknowledgement that ‘communication is between two (or more) people’, which means that ‘the responsibility for making communication work lies with both people’ (COM). They were frustrated that so ‘many, many interventions are aimed at making us fit the non-autistic norm’. Instead, as one respondent put it, ‘further research needs to be really clear about where the problem areas are – NOT the problem people. It sounds like a semantic difference, but it’s an important one’ (JUST). Some participants went so far as to suggest that the onus of responsibility should be shifted to the non-autistic majority: ‘I don’t believe the responsibility for change or compromise lies with the oppressed group. I think that as the dominant group, non-autistic people need to review societal structures, ideologies, power balances, and language’ (COM).

#### Subtheme 1.4: non-autistic researchers (and professionals) aren’t expert in being Autistic

Community members felt this pathologising narrative and its impacts were a direct consequence of not relying on Autistic experiential expertise in research: ‘we don’t want any more research that answers the questions NTs want answered. They tend to be obsessed with finding out how to “treat” us, rather than actually contribute to our wellbeing’ (H&W). Respondents described how there were ‘many delicate and controversial areas [of Autistic people’s lives] (JUST),’ which could be ‘easily understood by those with lived experience but are glossed over or dismissed’ (COM) by researchers and professionals, who ‘operate only on their observations and not lived experience’ (EDU).

They were resolute that ‘autistic people are the experts in themselves’ (EMP), yet repeatedly spoke about how individuals’ insights into their own lives were not valued or accepted by researchers. One participant went as far as to question whether universities ‘undervalue lived experience’ (EMP). Another participant felt that not being ‘treated as an equal partner’ (H&W) occurred even within this project’s consultation, suggesting that their views were ‘so often drowned out’ (COM).

### Theme 2: ‘we have so much to offer’

#### Subtheme 2.1: there are common threads but vast experiences

Respondents saw substantial value in the ‘vast experiences, expectations and understandings’ (H&W) across the Autistic and autism communities and felt that these insights could – and should – have much to offer. They discussed the value of thinking differently. One highlighted how Autistic people ‘are passionate advocates and very concerned to ensure Autistic people are treated fairly in all aspects of life’ (JUST). Some warned, however, these strengths were ‘not always appreciated or fully explored’ (EDU) or ‘recognised and valued’ (EMP), or even immediately apparent, especially if they were ‘accompanied by a difference in communication, which deter others from appreciating or uncovering them’ (EMP).

While some hoped that valuing Autistic strengths ‘would move us away from being seen through a lens of deficiency, and toward being seen through a lens of valued diversity’ (EDU), respondents also recognised that overemphasising strengths could also mean that ‘the challenges are overlooked and not understood’ (EMP). These challenges were manifold and often culminated from the negative experiences faced by Autistic people, ranging from being ‘convinced the world is unsafe as a result of having been a victim of crime on several occasions’ (JUST) to the hard realities of unemployment.

#### Subtheme 2.2: ‘we know what works for us’

Respondents also wanted greater understanding of ‘what a healthy, active life looks like, for us’ (H&W). They were sure that many Autistic people had abundant insights into their experiences and needs – ‘you know exactly what kind of support you want/need’ (EMP) – yet they were often not given ‘the time to be heard’ (H&W). Indeed, they were certain that ‘the way forward involves learning about autism and our own, unique presentation . . . and learning how to shape the world around us to suit our needs’ (H&W) so that ‘more supports will evolve and become inclusive’ (H&W).

Respondents were frustrated by the apparent gulf between research and practice. They spoke of wanting ‘flexible, responsive supports throughout life’ (H&W) and felt research had the potential to create supports that could ‘be individualised through novel methods’ (H&W). Some felt that researchers were at fault for this lack of ‘translation from medical into everyday speak’ (H&W), with professionals continuing to adopt ‘programmes that are not evidence-based’ (EMP) because best practice does not always ‘flow through’ from the research. One participant stressed that ‘whatever comes out of research needs to be actively and widely promoted and not just kept within the world of academia’ (EMP).

Yet, others felt, regarding education, that ‘teachers and schools need to be more proactive’ (EDU) in learning from research. Respondents in other groups agreed, worrying that too often ‘the research is ignored’ (EMP). Others still lamented that research just ‘often takes a long time’ (H&W) and that there often needs to be significant ‘political and societal will’ to deploy its findings (EDU).

#### Subtheme 2.3: the diversity of the Autistic community is a strength

Diversity was also taken to be a cause of this research-practice gap. Respondents acknowledged that ‘such a diverse range of people, experiences, families and communities could make it very hard to make conclusions’ (COM). Indeed, one respondent highlighted how Autistic people’s different support needs can ‘make it hard to set up support guides’ (EMP).

Some went further to suggest that ‘the problem lies with the ‘fear of the unknown’’ (EDU). Respondents wanted researchers ‘to understand that diverse perceptions and perspectives are not a threat . . . but can be a valuable contribution’ (EDU). Others agreed that ‘broadening understandings of autism and valuing the contribution of autistic people benefits everyone’ (EMP) and would give Autistic people ‘more power to contribute positively’.

In addition to the breadth of Autistic experience, respondents also spoke of the strong ‘sense of belonging’ (H&W) that can connect Autistic people despite all their differences. Respondents spoke of a need for a space where ‘all our strengths, interests and natural motivations are . . . encouraged and explored’ (COM) and new information and insights ‘are shared among others’ (H&W). While they acknowledged that disagreements between Autistic community members were inevitable, they also felt that, ‘on the whole, I see more consideration, more listening, and more respect in Autistic communities’ (COM).

### Theme 3: it’s time to take Autistic people seriously

#### Subtheme 3.1: ‘nothing about us without us’

Respondents were committed to ‘the idea of ‘nothing about us without us’’ (COM). They felt strongly that ‘a lot of Autism research is full of blind spots and false assumptions, because a lot of autism research . . . is still failing to fully incorporate Autistic perspectives’ (EMP). Some felt that ‘neurotypical people have been monopolising the conversation for way too long’ (COM). They were therefore resolute that it was time ‘to co-produce our knowledge . . . when we add to a concept, we don’t just jam it in and assume it’s right; we listen and collaborate to get a “right” that we all have (when possible)’ (COM). They appealed to researchers not to research something simply because it is ‘easy to design the study and isolate dependent and independent variables’ (COM). Instead, they encouraged researchers to ‘collaborate with real people and service providers to find the key topics . . . then find a way to make the research as robust as possible’ (COM).

Respondents wanted to see greater Autistic involvement ‘at every stage of research’ (JUST) and also in ‘any education or training’ (H&W). Ideally, they felt that Autistic people should be ‘driving the narrative’ (H&W), emphasising that research could help push ‘towards acceptance and understanding of neurodivergence’ (COM) and would ‘likely lead to more fulfilling lives’ (EMP).

Some spoke of the need to ‘include family or support networks, not just the Autistic individual’ (H&W), ‘if it is to be useful and meaningful’ (COM). Others, however, warned that this broader involvement risked diluting ‘the voice, perspective and lived experience of the autistic community’ (COM). One non-speaking participant felt that ‘researchers need to keep in mind that equally weighting the voices of non-autistic and autistic people will lead to research that, at least partway, prioritises goals of *allistic* people over those of autistic people’, especially for non-speaking Autistic people who ‘are ignored and excluded in so many spaces’ (COM).

#### Subtheme 3.2: value ‘our information, our knowledge, our insights’

Respondents wanted researchers to recognise that Autistic people ‘can contribute incredibly if given the opportunity, even if it looks a little different’ (EMP) with potential to discover and contribute ‘weird and wonderful solutions to problems’ (H&W).

Respondents also noted that valuing ‘a range of Autistic perspectives’ (COM) requires greater recognition of how Autistic people communicate. They were aware that ‘people still get mad because I don’t say things nicely enough’ (COM). They suggested that researchers should ‘try not to get offended’ (COM) but instead ‘presume it comes with good intentions, respect and a desire to improve the understanding and lives of Autistic people’ (COM). Interactions based on mutual respect with ‘the right people’ (EMP) should ensure that they feel safe offering such insights given the all-too-frequent experiences they may have had ‘being ignored or questioned on everything’ (COM).

#### Subtheme 3.3: community members want ‘more power to contribute positively’

It was clear that Autistic respondents and their allies felt they had an important role to play in the future of autism research and saw this as critical to ensuring their own wellbeing and that of others in the Autistic community. They wanted research ‘with our voices centred, our experiences validated and with our control’ (H&W). Nonetheless, some were sceptical that greater power-sharing would come in research, suggesting that ‘it is a privilege they [non-autistic people] are unlikely to want to give up’ (COM). They noted that few researchers were ‘open-minded enough’ (H&W), suggesting ‘[non-autistic] people don’t want to change, are afraid of any change, or going outside their comfort zone’ (JUST).

Others, however, were more hopeful that ‘genuine participation could occur’ (JUST). These respondents noted the need to ‘address the gatekeeping barriers’ (H&W) through advocacy ‘at the highest level’ (JUST). They wanted to ‘work together to address myths and stereotypes’ (JUST) and to ‘co-produce knowledge’ (COM). They welcomed researchers ‘that are driven in this field and begin to pass on their gained/educated knowledge’ (JUST).

Respondents also stressed that this collaborative approach should not feel threatening to researchers (see also subtheme 2.3): ‘I’m not bringing in new knowledge because I want your job, your friends, a raise, or because I’m challenging your authority. I’m bring[ing] new knowledge so we can all benefit’ (COM). Respondents were encouraged by encounters with respectful and affirming researchers, who they felt often made an enormous difference to their experience ( ‘it’s a totally different experience when you finally encounter someone who has a clue’; H&W), and could have far-reaching effects: ‘feeling looked after, listened to and cared for has the potential to make a profoundly positive impact on someone’s life – their mental health, self-esteem and their confidence’ (H&W).

## Discussion

The community members involved in this study resoundingly agreed that autism research could play a powerful role in helping to shape good Autistic lives, but they felt that if it was to do that effectively, then it needed to change in some crucial regards.

Community members were frustrated with what they perceived to be the pathologising discourse – of being ‘broken’– that often accompanies the conventional paradigm of autism. They worried these conceptualisations were damaging to Autistic people’s self-worth and family wellbeing, and that these views were present across society, including in the research process. More worryingly still, they saw what they called these ‘less than human’ attitudes (Goffman, 1990; see Botha & Cage, 2022) extending well beyond the research community, impacting how Autistic people were supported, perceived, and responded to at school, in workplaces, and in community settings. These perspectives are well reflected in the developing literature. Botha and Cage (2022) showed that many conventional autism researchers are likely to describe autism and being Autistic in ways that are experienced as powerfully negative and critical by Autistic people themselves.

Such negative attitudes pose deep challenges. They may contribute to the fact that Autistic people, including children and young people, report seeing themselves as being ‘different’ from people in a negative way (Shattuck et al., 2014; Williams et al., 2019), labelling themselves as a ‘freak’ or as ‘having a bad brain’ (Humphrey & Lewis, 2008), and wanting just to be ‘as normal as possible’ in order to fit in (Baines, 2012; Cribb et al., 2019). At worst, these negative attitudes about being Autistic can have damaging effects on Autistic people’s self-esteem and self-concept (van der Cruijsen & Boyer, 2021; Williams et al., 2019) and on their mental health (Cage et al., 2018; Cooper et al., 2017). Even if it does not go this far, many Autistic people report facing the often-relentless burden of making themselves understood to neurotypical people, rather than neurotypical people feeling a need to make themselves understood to them. This has become known as the double empathy problem (Milton, 2012).

At the heart of this challenge is a sense that Autistic people have insights to share with non-autistic people. Failing to attend to that properly leads to constraints in our understanding of autism. Our respondents were concerned that the ‘blind spots and false assumptions’ in current ways of thinking precluded autism research from making a meaningful difference. Again, they had good reasons to feel this way. It is becoming clear that there are ‘blind spots’ in research, or cases of research areas not being pursued because the neuronormative lens of the prevailing paradigm leads scientists to characterise some areas as not worth studying. This leads to so-called ‘undone science’ (Hess, 2016) or to ‘false leads’ (see Bottema-Beutel et al., 2023)

One such example of false leads in autism research is the focus on ‘diminished social motivation’ (Chevallier et al., 2012), which suggests that Autistic people’s ‘social deficits’ exist because they fundamentally ‘lack the motivation or capacity to share things psychologically with others’ (Tomasello et al., 2005, p. 723). This theoretical framing has led to a swathe of research studies focused on identifying the biological substrates for ‘abnormal’ reward processing (e.g. Bottini, 2018; Clements et al., 2018). Yet, recent critiques of this hypothesis (Jaswal & Akhtar, 2018) have emphasised that social interactions are shaped by *all* parties, which means that non-autistic people have as much part to play in any challenges associated with Autistic people’s interactions as Autistic people (Milton, 2012) – just as our respondents themselves attested. Autistic perspectives also resoundingly contradict the very premise of this influential theoretical account, by showing that Autistic people *want* to connect with others (e.g. Pellicano et al., 2021) and can and do have fulfilling connections with friends, family and lovers, especially with those who accept them for who they are (e.g. Crompton et al., 2020; Morrison et al., 2020).

Despite their deep frustrations with current autism research, community members were nevertheless resolute about how autism research could change so that it can be ‘useful and meaningful’. They wanted to ‘change the narrative’ from viewing Autistic people, not as a collection of ‘deficits’ needing to be ‘fixed’, but as unique and worthwhile individuals. They were adamant that reframing autism in this way did not necessarily mean that individual challenges did not exist (den Houting, 2019), but that greater emphasis was needed on understanding the ‘individual-in-context’ (Woodly, 2022) and, importantly, on identifying and implementing contextually-relevant *responses* that could positively impact Autistic lives. These views connect with the neurodiversity paradigm (den Houting, 2019; Kapp, 2020; Pellicano & den Houting, 2022; Singer, 1998; Walker, 2012), which stresses the need to value Autistic lives and to look beyond the individual by focusing on the context *and* the interaction between contextual and individual factors. Also, rather than seeing the variability among Autistic people as a ‘nuisance factor’, the neurodiversity paradigm urges us to see strength in diversity (Fletcher-Watson, 2022) – a sentiment also emphasised by our respondents.

Our respondents’ views are consistent with mounting calls for a ‘more humanising autism science’ (Botha & Cage, 2022, p. 17; see also Lory, 2019; Michael, 2021), partly through adopting more reflective praxis (Botha, 2021; Tan, 2023; Thompson-Hodgetts, 2023), and partly through addressing how Autistic children, young people, and adults are regarded and treated throughout the research process (Cascio et al., 2020), especially in intervention studies (Bottema-Beutel et al., 2020; M. Dawson & Fletcher-Watson, 2022). There is also innovative work that demonstrates that the perceptions, attitudes and behaviours of *non-autistic* people might be one important source in the breakdown of reciprocity within Autistic – non-autistic interactions (Chown, 2014; Milton, 2012; see Davis & Crompton, 2021, for review) and highlights the importance of meeting Autistic people (at least) half-way.

Above all of this, our respondents were insistent that it was time for Autistic people and their allies to be formally engaged as partners in the research process. Historically, Autistic people have been left out of the decision-making processes around research (Pellicano et al., 2014a, b). This systemic exclusion from knowledge production about autism – one form of epistemic injustice (Fricker, 2007) – was keenly felt by our respondents. There has, however, been a gradual shift towards developing rigorous methods to work *with* members of the Autistic and autism communities in both biomedical (Heraty et al., 2023; Pellicano, 2020) and applied (Fletcher-Watson et al., 2019; Jose et al., 2020; Nicolaidis et al., 2019) research, *and* concomitant increases in the adoption of participatory approaches in autism research (Tan et al., 2024).

Such involvement requires substantial commitment by researchers to listen to, and learn from, the experiential expertise of a diverse range of Autistic people (Collins & Evans, 2002; Milton, 2014), and to be willing to share power with community members (Nicolaidis et al., 2019). This has been our intent in this preparation of this article itself, which is the product of a team of Autistic and non-autistic researchers and which involved continuous consultation and reflection throughout its design and production. Sharing power is rarely straightforward, however – and even well-intentioned participatory studies can struggle to address power differentials between researchers and non-researchers, often leaving community partners feeling excluded^3^ (den Houting et al., 2021). Tan et al.’s (2024) systematic review demonstrates that, of the studies reporting community involvement in autism research, most were at the consultative rather than the collaborative level, reflecting only partial involvement.

Beyond the difficulty with power-sharing, researchers might also find it challenging to conduct participatory autism research, due to additional time, resources and funding often required and the lack of a standard approach (den Houting et al., 2021; Pellicano et al., 2014a; Pickard et al., 2022; Raymaker & Nicolaidis, 2013). Support and training are needed for researchers to feel more confident in appreciating Autistic people’s distinctive expertise (Milton, 2014), and in developing ‘epistemic fluency’ (Markauskaite & Goodyear, 2016), or the ability to flexibly combine different forms of expertise and different ways of knowing. Being exposed to a diverse range of views and experiences – precisely as our respondents suggested – as opposed to more selective community involvement (Russell et al., 2018; see also Ocloo & Matthews, 2016), might be one way of promoting such fluency. Bridging the ‘epistemological divide’ (Ward et al., 2010) in this way should lead to new co-constructed ways of thinking about autism and good Autistic lives (Chapman & Carel, 2022; Pellicano & Heyworth, 2023) and more effective, meaningful research that ‘contributes to our wellbeing’.

### Limitations

This research has limitations. The secondary data were both de-identified and de-linked, meaning that it was not possible consistently to attribute quotes to people’s connection to the Autistic/autism communities. Relatedly, while the focus group members had a range of experiential and professional expertise and the number of Autistic people (60%) far exceeded the percentage of Autistic people previously included in research priority-setting exercises (9% of samples across six studies; Roche et al., 2021), the limited demographic data available meant that we do not know how representative the sample was in terms of socioeconomic status, racial/ethnic background or other key characteristics. While the AARC made extensive efforts to select focus group members with a diverse range of relevant perspectives, and the current findings echo the sentiments from Autistic people and parents of Autistic children across different backgrounds (e.g. Malone et al., 2022), there may well, of course, be perspectives not captured in the process.

## Conclusion

We too often forget the context within which autism research takes place. Autistic people are subject to significant injustices and inequalities during their lives, and they understandably feel that autism science has so far done relatively little to redress them. In this study, drawing on the deliberations enabled by the AARC, we were able to present a clear picture of the manifold ways in which Autistic and autism community members currently find the conduct of autism research lacking. The challenge now, of course, is to change it.

## Notes

1. We have chosen to capitalise the word Autistic to indicate a proper adjective, which reflects its status as a robust and valued identity and shared community, similar to the Deaf community.

2. The Justice priority referred to measures that can be taken to prevent discrimination, exploitation, violence and abuse of Autistic Australians, and ensure equity in accessing justice.

3. This finding also highlights the potentially harmful consequences that can arise from tokenistic participatory practices (e.g. Brett et al., 2014; Omeni et al., 2014).
